# Exploring the interplay of family dynamics and pregnancy supplement adherence among married women of reproductive age: a qualitative study from rural Bangladesh

**DOI:** 10.1136/bmjopen-2025-115088

**Published:** 2026-05-12

**Authors:** Pragna Paramita Mondal, Mary de Boer, Atiya Rahman, Nazrana Khaled, Eleonor Zavala, Rezwanul Haque, Hasmot Ali, Towfida Jahan Siddiqua, Kaosar Afsana, Parul Christian, Andrew L Thorne-Lyman, Anna Kalbarczyk

**Affiliations:** 1 James P Grant School of Public Health, BRAC University, Dhaka, Bangladesh; 2 Department of International Health, Johns Hopkins Bloomberg School of Public Health, Baltimore, Maryland, USA; 3 JiVitA Bangladesh, Rangpur, Bangladesh

**Keywords:** Pregnancy, Nutrition, PUBLIC HEALTH

## Abstract

**Objectives:**

To examine how household members, community health research workers (CHRWs) and broader social networks influenced pregnant women’s capabilities, opportunities and motivations to consume a daily balanced-energy protein (BEP) supplement or a multiple micronutrient supplement (MMS) in the context of an effectiveness trial in rural Bangladesh.

**Design:**

In-depth interviews, group interviews, focus group discussions, thematic analysis using the Capability, Opportunity, Motivation-Behaviour (COM-B) framework.

**Setting:**

Gaibandha, Bangladesh.

**Participants:**

Women (n=32) who had completed participation in the TARGET-BEP randomised trial, their husbands (n=13) and mothers-in-law (n=13), who participated in 13 group interviews, and CHRWs (n=39) who participated in six focus group discussions.

**Results:**

*Capability* to adhere to BEP and MMS was strengthened when family members understood the value of supplements and actively supported supplementation. Children emerged as unexpected facilitators, reminding mothers to consume supplements and tracking intake. *Opportunity* to use supplements consistently was enhanced by women’s educational attainment and the availability of household resources. Finally, *motivation* to take the supplements was influenced by many actors including neighbours, who could offer support but also often transmitted rumours and taboos, and CHRWs, who adeptly adapted adherence messages to the local context and to women’s specific concerns.

**Conclusions:**

To improve antenatal supplement adherence and maternal–infant health in Bangladesh and similar contexts, pregnancy nutrition programmes should move beyond the woman-as-sole-agent paradigm by: (1) co-designing messages for husbands, mothers-in-law, children and neighbours in conversation with effective community health workers, such as those working in the TARGET-BEP trial; (2) equipping community health workers with flexible, family-engaging counselling strategies; and (3) complementing women’s education gains with gender-transformative and family-inclusive interventions.

**Trial registration number:**

ClinicalTrials.gov NCT05576207

**STRENGTHS AND LIMITATIONS OF THIS STUDY**

- Incorporates multiple perspectives, including women, their husbands, mothers-in-law and community health research workers, allowing for triangulation across household and community actors.
- Applies the Capability, Opportunity, Motivation-Behaviour (COM-B) framework embedded within the bioecological model to guide a theory-informed analysis.
- Draws data from a randomised-controlled trial, which both enhances context-specific credibility of the findings but may limit transferability due to the free provision of supplements and close adherence monitoring in the trial setting.
- Although qualitative interviewers were external to the trial implementation team, participants may have still framed their responses in ways perceived as favourable to the trial organisation, which has a long-standing presence in the community.
- Interviews were conducted after completion of the trial, which may have risked introducing recall bias of adherence behaviours.

## Introduction

 Antenatal service delivery in many low and middle-income countries often operates through a biomedical lens, focusing narrowly on the woman and neglecting the multiple overlapping socioeconomic, cultural and familial contexts in which she is embedded.^1^ In reality, women’s health and dietary behaviours are negotiated within families, including with husbands and mothers-in-law living in the same household. These family members can act as gatekeepers of household resources, norms and expectations, influencing women’s ability to adopt new practices or access services.^1 2^ In some contexts, lack of financial autonomy can restrict women’s ability to pay for transportation or health services, while gender norms can also limit mobility and agency.^3 4^

In pregnancy, these dynamics can be particularly influential as norms around care and diet become heightened.^5 6^ The 2016 WHO guidance for antenatal care suggests greater contextualisation of service delivery,^7^ which can help women better navigate these complex intrahousehold and community-level dynamics, but few countries have adapted their practices accordingly.^8^ According to the Bangladesh Demographic and Health Survey,^9^ less than 40% of women had at least four antenatal care (ANC) visits, with the lowest rates among rural women. Instead, pregnant women living in rural or under-served areas are typically reached through community-level courtyard meetings, facilitated by governmental or non-governmental organization (NGO)-supported community health workers (CHWs), as well as receiving one-on-one individual counselling, either at home or in health facilities.^10^ When well-structured, funded and inclusive of family members, these interventions have improved nutrition knowledge and, in some cases, behaviours.^11^ However, challenges remain in maintaining quality, increasing scale, and ensuring financial sustainability.^12 13^

Shifts in gender and generational roles are also redefining the landscape of family and community influence. Increased access to education, evolving economic pressures and changing gender expectations are reshaping traditional family hierarchies globally and within South Asia.^14 15^ In Bangladesh, women’s autonomy in health decision-making is linked with higher education and employment,^16 17^ but family dynamics remain key. Patrilocal marital practices and traditions of purdah, often reinforced by elder women family members, constrain women’s decision-making power.^18^ Recent research in Bangladesh also highlights the influence of actors beyond the immediate household on health and nutrition behaviours.^19 20^ These actors include neighbours, community leaders, religious leaders and CHWs, who mould norms around pregnancy diet, supplement use and care-seeking pathways^1^,^4^, ^3^,^6^ thereby influencing women’s adherence to recommended antenatal nutrition practices.^1 4 6^ Understanding these evolving dynamics of power and influence is essential for designing interventions that are responsive to the realities of women’s lives.

Maternal nutrition interventions such as daily iron-folic acid, multiple micronutrient supplements (MMS) and balanced energy protein supplements (BEP) are most effective if consumed regularly as recommended throughout pregnancy.^21 22^ But adherence is not just a matter of access or individual awareness^22^,^24^; it depends on both individual and household acceptance and perceived appropriateness.^23 25^ Most research focuses on individual knowledge and behaviours, however, with less attention paid to these relational influences or household dynamics.^26^

The TARGET-BEP randomised controlled effectiveness trial (ClinicalTrials.gov NCT05576207) provided a unique opportunity to explore factors influencing acceptability and adherence to BEP and MMS, including women’s perceptions of the supplements, household decision-making dynamics and community norms surrounding maternal nutrition. The trial assessed the effect of untargeted vs targeted antenatal BEP supplementation alongside and compared with MMS alone as control on the outcomes of birth weight, low birth weight and small for gestational age in rural Bangladesh.^27^ BEP supplements and the MMS control were delivered monthly by project-hired female community health research workers (CHRWs) who were also tasked with providing antenatal counselling to the enrolled study participant and her family members and encouraging high adherence. CHRWs also collected data on tablet and sachet counts and tracked pregnancy outcomes. The monthly interactions between pregnant women, their family members and the CHRWs provide an opportunity to investigate how family and community dynamics influence BEP and MMS use in rural Bangladesh. While prior qualitative studies have explored knowledge, attitudes and acceptability of antenatal supplements, few have analysed how these dynamics shape adherence. By examining supplement use within an effectiveness trial, this study provides a theoretically informed, implementation-sensitive perspective to guide gender-responsive and socially aware nutrition programme design.

## Methodology

### Study design

This qualitative study aimed to examine the influence of familial, health worker and community networks on women’s uptake of the nutritional supplements in the TargetBEP trial. Women from each of the four arms of the TargetBEP trial were identified for in-depth interviews (IDIs) focusing on their experiences with the supplements. Group interviews (GIs) were conducted as dyadic interviews with a sub-sample of these women’s family members (husbands and mothers-in-law) to assess the ways in which household dynamics influenced women’s experience of and engagement with supplementation.^28^ Finally, CHRWs participated in focus group discussions (FGDs) to explore how they had supported women and families to be involved in the trial and maintain high adherence to supplements.

### Study site and the TARGET-BEP trial

The JiVitA study site is located in a rural area of Gaibandha district, in northwestern Bangladesh. The site was selected based on maternal and child health indicators that were similar to rural Bangladesh and neighbouring countries.^29^ Maternal and child nutrition research has been conducted at the site since 2000, including a large trial of MMS from 2008 to 2014, making MMS a familiar intervention to most study participants and their families. All trials in the intervention area have been implemented and monitored by cadres of female CHRWs, previously called field distributors, recruited from the local communities. Past research at the study site has documented the trust community members place on CHRWs as reliable sources of information on pregnancy nutrition and good antenatal practices.^30^

The current trial, TARGET-BEP, ran from 2023 to 2025, and data for the qualitative study were collected during September and October 2024. The trial compared universal BEP supplementation to two anthropometry-based BEP targeting methods and a control of MMS. The locally produced BEP was a lipid-based, micronutrient-fortified supplement (‘PregaVita’) offered in unflavoured or malai (clotted cream) flavoured rice-lentil or chickpea (added later) forms and tested among women in the intervention area for acceptability.^23^ The trial had four arms: (1) universal MMS (control), (2) universal BEP, (3) targeted BEP for women with pre-pregnancy body mass index (BMI) <18.5 kg/m² and MMS for all other women and (4) targeted BEP for pre-pregnancy BMI<18.5 kg/m², with all other women receiving MMS, and those who subsequently experienced inadequate gestational weight gain switched from MMS to BEP until the end of pregnancy.

In the TARGET-BEP trial, CHRWs were responsible for providing women with both nutritional supplements (BEP and the MMS control) every month, monitoring their adherence over the telephone at mid-month, and collecting used packets to measure adherence. They also provided counselling to women on the nature of the products and their benefits for pregnant women, different ways to consume them to increase their palatability, suggestions on managing perceived side effects and pregnancy symptoms, and encouragement to both trial participants and their family members to support high adherence, including discouraging women from sharing the supplements with others.

### Study participants and sampling

Participants for IDIs were systematically identified from among women who had completed trial participation and delivered a live infant within the previous 6 months. Participants were sampled to represent a diversity of trial characteristics including allocation arm, low and high adherence, low and high household socioeconomic status and residence in the eastern and western parts of the study area to capture the study area’s known east-west gradient in rurality.^29^ Low adherence was defined as the bottom quartile of supplement consumption and high adherence as the top quartile of consumption, based on completed packet counts; high household socioeconomic status (SES) was defined as the highest quartile of a within-study calculated asset index and low household SES as the lowest quartile.^31^

Additionally, GIs were conducted in a dyadic format with 13 husband and mother-in-law pairs from a subsample of the women interviewed. Husbands and mothers-in-law are key health and nutrition decision-makers within Bangladeshi culture, and the GI technique allowed for dialogue exploring the perspectives of both individuals involved in a decision-making process.^28^ The families selected for GIs were identified to represent diversity across the three BEP intervention arms (2, 3 and 4), household socioeconomic status, and geographic location (see table 1, below).

**Table 1:** Family sample composition

	East n=7		West n=6	
	SES low	SES high	SES low	SES high
	**Arm 2: Universal BEP**			
Adherence low	1		1	
Adherence high		2		1
	**Arm 3: BEP targeted on low BMI**			
Adherence low				1
Adherence high		1	1	
	**Arm 4: BEP targeted on IGWG**			
Adherence low	1		1	1
Adherence high	1	1		

BEP, balanced energy protein; BMI, body mass index; IGWG, inadequate gestational weight gain ; n, number of families; SES, socioeconomic status.

Six FGDs were conducted with 39 CHRWs (6–7 per FGD). CHRWs were selected to include those who had interacted with the women selected for IDIs, had over 6 months of on-the-job experience, and had worked in one of 6 geographic units from which study participants were sampled.

Sample size was determined based on previous empirical studies suggesting that 9–17 interviews or 4–8 FGDs are typically sufficient to achieve thematic saturation in qualitative studies.^32^

### Data collection

Four female qualitative interviewers (QIs) with anthropology or social science degrees were trained by three researchers from BRAC University’s James P. Grant School of Public Health (JPGSPH) and one PhD candidate at Johns Hopkins University, with support from the JiVitA field staff. The 6-week training covered qualitative research techniques, study background and objectives, field guide pre‐testing, and human subjects research ethics. Structured interview guides were developed to capture four key constructs: perceptions and acceptability of the BEP and MMS supplements, barriers and facilitators of adherence to the supplements, appropriateness of targeting, and hypothetical costs of the supplements. These constructs have been established by implementation scientists as crucial for programme success.^33^ Selected probes were suggested in the interview guides, while QIs were also trained to use repetition, paraphrasing, simple affirmations and active listening techniques to elicit further information from respondents. All interview guides and materials were pre-tested and adapted based on the pre-testing.

All IDIs and group discussions were facilitated by the trained QIs. With participants’ consent, sessions were audio-recorded; when recording was not permitted, detailed field notes were taken. Transcription and translation of the recordings and notes were completed by the QIs, and the resulting transcripts were reviewed and cross-checked by research supervisors to ensure accuracy and consistency. Data collection took place from 8 September 2024 to 10 October 2024, followed by 2 months of transcription and translation.

### Data analysis

In the qualitative data analysis, the team applied both deductive approaches—relying on predefined and theory-driven codes drawn from the Proctor Implementation Outcomes framework concepts^33^—and inductive approaches—allowing codes to emerge from the data; the team moved back and forth between the two approaches during the coding process and through other phases of the analysis.^34^

Initially, each team member re-reviewed the same transcript, generated inductive codes, and memoed ideas related to the codes and emerging themes. The team met to compare progress and draft a common codebook (Codebook included in online supplemental materials S1). The team discussed any discrepancies in coding to develop a common approach.

The interviews were coded in Atlas.ti for the web (V.9.22.0-2025-08-26). The team met weekly to discuss memos, emerging themes, and questions that arose during coding. Partway through the analysis, the team reviewed one another’s coded interviews once again and discussed any remaining discrepancies in the coding approach.

To facilitate comparative analysis and triangulation,^35^ the team developed tabular summaries triangulating the descriptions of key themes across the different family members (participant, husband, mother-in-law). Codes were subsequently grouped into two main clusters: the first cluster related to adherence (pregnancy-related morbidities, acceptability, delivery-related characteristics, individual factors, socioeconomic factors based on an adherence framework^36^ and the second cluster related to targeting (appropriateness, trust and access)). We used the Standards for Reporting Qualitative Research (SRQR) reporting guideline^37^ to draft this manuscript, and the SRQR reporting checklist when editing (included in online supplemental materials S2).

### Analysis framework

The analysis of the family member dynamics relied on a modified version of the Capabilities, Opportunities, Motivations and Behaviours (COM-B) framework,^38^ incorporating elements of the Bronfenbrenner bio-ecological model.^39^ In COM-B, behaviour arises from the interaction of three components: capability, opportunity, and motivation. While COM-B addresses individual-level and some contextual influences, it does not fully account for how individuals are embedded within multiple environmental systems.^40^ This perspective was added by incorporating Bronfenbrenner’s bio-ecological model, which envisages individuals as nested within interrelated systems, including microsystems such as home, family, school, and peer networks; mesosystems that link these microsystems (e.g., parent-teacher conferences, health service interactions); and the macrosystem of overarching cultural norms and values.^39^ Each microsystem has distinct characteristics that shape how individuals engage with people, objects and institutions in their environment, a process that is further influenced by an individual’s characteristics (such as age, gender or health status).^41^

**Figure 1:**
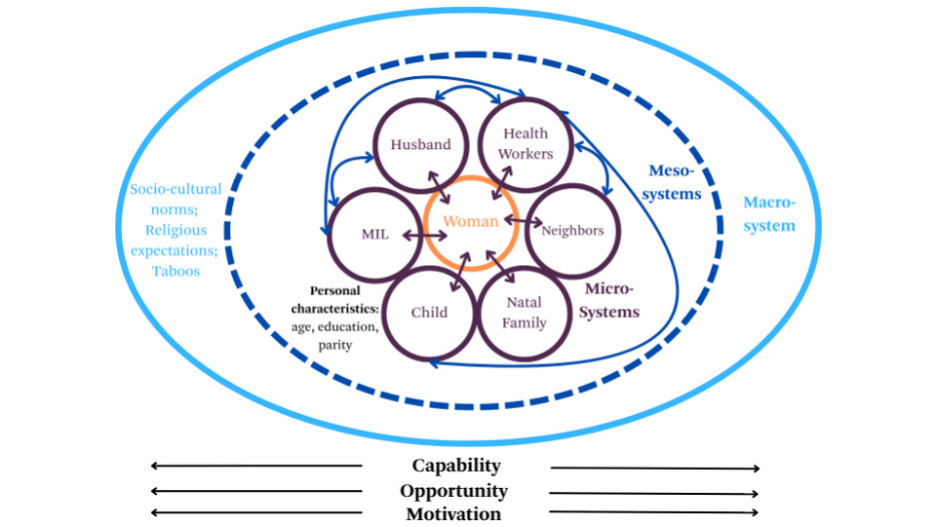
Authors’ conceptualisation of the Capability, Opportunity, Motivation-Behaviour Framework, embedded in Bronfenbrenner’s bioecological model.

In combining these models, we envisioned capability, opportunity and motivation as aspects that cut across the different micro, meso and macrosystems. Microsystems were made up of the different dyadic interactions between the woman and the other actors in her environment, influenced by her own personal characteristics, while mesosystems were the interactions between the different dyads—an important area of focus and inquiry for this study. Macrosystems remained unchanged. All influenced the woman’s capability, opportunity and motivation for uptake and consistent use of antenatal supplements. Our combination model is presented in figure 1.

### Reflexivity statement

This study was conducted by a multidisciplinary team of researchers with backgrounds in public health, nutrition and anthropology, working collaboratively across institutions in Bangladesh and the USA. Team members’ positions in academic institutions and research organisations offered both strengths and challenges throughout the research process. The research team included both local researchers who were closely familiar with the sociocultural context of the study area and international collaborators with experience designing and evaluating interventions related to maternal nutrition and health in diverse settings. Several co-authors were involved in the broader TARGET-BEP trial design and oversight, while others were engaged only in the qualitative component and not responsible for day-to-day trial implementation. Members of the study team, JPGSPH and Johns Hopkins University, had longstanding relationships with JiVitA. These relationships facilitated community access, trust, and rapport with the study participants and allowed for deep contextual insight and respectful engagement with participants. At the same time, the study team remained aware of the potential for social desirability bias, particularly given JiVitA’s established presence and reputation. To mitigate this bias, data collection was done by trained QIs external to the trial implementation team. The interviewers were introduced as independent researchers conducting a separate qualitative component. The study team continuously reflected on how their professional backgrounds, assumptions, perceptions and the power dynamics inherent in cross-cultural, interdisciplinary research could influence data interpretation. During analysis, the team actively engaged in team-based coding and regular analytic debriefings and weekly meetings to challenge personal perceptions and assumptions and to surface alternative interpretations for ensuring reflexive awareness and inclusion of diverse perspectives.

### Findings

#### Participant characteristics

The 32 women in the study sample had a mean age of 25 (SD±5) years. Women had a mean of 8 (±4) years of education. Husbands were most likely to work in agriculture (n=7, 54%) or have a small business (n=2, 13%). Husbands had a mean age of 32 (±6) years and 10 (±5) years of education. Mothers-in-law had a mean age of 53 (±8) years, and all interviewed mothers-in-law were uneducated. CHRWs included in the FGDs had a mean of 20 (±5) years of experience as a JiVitA health worker.

The 13 families covered all three BEP arms (targeted and untargeted), as well as the different SES and adherence combinations across the TARGET-BEP region (table 2).

**Table 2:** Family characteristics, n=number of families

Family characteristic	Overall n=13 (%)
BEP targeting schema	
Universal	5 (38%)
Low BMI	3 (23%)
IGWG	5 (38%)
Geographic cluster	
East	6 (46%)
West	7 (54%)
Adherence	
High	6 (46%)
Low	7 (54%)
Socioeconomic status	
High	5 (38%)
Low	8 (62%)
Maternal education	
None	1 (8%)
Primary	2 (15%)
Some secondary	9 (69%)
Post secondary	1 (8%)
Maternal age (years)	
16–20	4 (31%)
21–25	5 (38%)
26–30	3 (23%)
31–35	1 (8%)
Paternal education	
None	0 (0%)
Primary	6 (46%)
Some secondary	3 (23%)
Post secondary	4 (31%)
Paternal age (years)	
16–20	0 (0%)
21–25	2 (15%)
26–30	5 (38%)
31–39	6 (46%)
Mothers-in-law education	
None	13 (100%)
Any	0 (0%)
Mothers-in-law age (years)	
40–49	5 (38%)
50–59	5 (38%)
60–69	2 (15%)
70–79	1 (8%)

.BEP, balanced energy protein; BMI, body mass index; IGWG, inadequate gestational weight gain.

#### Capability

In the COM-B framework, capability is defined as having the knowledge, skills and abilities to engage in a behaviour.^36^ For the purposes of this analysis, we defined capability as the individual’s ability to consistently adhere to daily supplement intake (MMS or BEP), including how this was influenced by her surrounding family and community dynamics. Family members played a key role in shaping women’s capability by either reinforcing knowledge about supplements, either facilitating the daily consumption routines or constraining women’s ability to take them.

Knowledge of maternal nutrition practices was high among both study participants and family members. The family recognised that good nutrition during pregnancy can contribute to the baby’s development and mother’s health and that taking supplements could help fill dietary gaps. One husband stated that he had encouraged his wife to consume BEP,

The nutrition we get in our area (community) doesn't fulfill all the needs. And when a baby is conceived, extra nutrition is required. That’s why [BEP] is needed. (Family K: Husband, post-secondary education)

In this way, husbands often facilitated capability by translating their awareness of nutrition into practical encouragement of daily supplement intake. When husbands felt they lacked the knowledge to support supplementation, many actively sought it out, reaching out to health providers or trusted personal contacts.

In another family, the wife noted that men like her husband, who worked in a tailoring shop in the market, were often too busy to be knowledgeable about or engaged in women’s food choices. Nonetheless, he encouraged her ‘not to waste’ the supplement, because he knew and trusted the JiVitA organisation:

I know about JiVitA Bangladesh. Whatever they give is good. (Family I: Husband, junior secondary education)

Likewise, in the same family, while the woman’s mother-in-law expressed some hesitation about BEP, she shared that she had not objected to the woman taking the supplement because she, toom trusted JiVitA. This trust in the implementing organisation indirectly supported women’s capability, as family members who lacked detailed nutritional knowledge still legitimised supplement use. Overall, despite differing levels of knowledge about and engagement in their wife’s or daughter-in-law’s supplements, most participating families were familiar with the supplements and expressed knowledge of their potential benefits, with many taking steps to actively facilitate and encourage women’s capability to consume them.

There were, however, some exceptions. CHRWs in one focus group reported that women’s capability to consume supplements was at times constrained by mothers-in-law who believed that supplements could lead to a large baby and the need for caesarean section. As one CHRW described,

Her mother-in-law doesn’t let her eat. She (Mother-in-law) says that the baby will get fat, and the woman will need a C-section… The woman secretly eats (the supplements) without the mother-in-law’s knowledge and tells me, “We are poor people. Please don’t tell my mother-in-law that I’m taking these. Just say you’re here to check my weight and blood pressure.” (FGD TBC-05, Participant 5)

Here, the mothers-in-law acted as gatekeepers of household health practices and decision-making, limiting women’s capability to openly adhere to supplements. In these situations, CHRWs reported that husbands typically deferred to their mothers. Notably, while multiple CHRWs described this dynamic, no participants directly reported such situations. Rather, it was more common for husbands to dismiss their mothers’ beliefs, saying, for example,

It’s not like that, our mothers don’t understand much. They are from the village, not very well-informed. (Family J: Husband, primary education)

Likewise, women often shared that their mothers-in-law were disconnected from their experiences of supplementation.

Family dynamics could further shift when women returned to their natal homes during late pregnancy. Women in the study area frequently travel to stay with their family of origin in their second or third trimester, where they can experience more positive, supportive environments, with families of origin encouraging the women to take supplements. As one CHRW recounted,

The woman’s mother understands the importance of this packet. Her mother keeps saying that she needs to eat the packet. Her mother said, ‘Your mother-in-law, no matter what she said, you should eat this. (FGD TBC-05, Participant 5)

This change of residence often allowed women greater autonomy over health behaviour and decision-making and thereby strengthened their capability to maintain adherence. One husband, whose wife had returned to her father’s house in the seventh month of pregnancy without him, noted he knew little of her later pregnancy, as it occurred in his absence. However, he expressed confidence in his wife’s capability to make her own decisions regarding nutritional supplementation. His confidence was partially attributed to her educational background. As he mentioned,

She is educated too. She is more educated than me. She understands better. (Family E: Husband, secondary education)

In addition to senior family members, children also posed a challenge for women’s capability. Children’s curiosity as well as attraction to the supplement as a ‘snack’ sometimes risked diverting it from the pregnant woman. CHRWs messaging and counselling materials, including brochures, emphasised that the supplements were not to be shared. As a result, in response to this pressure from their children, women might ‘sneak the food or hide it’ (FGD TBC-06, Participant 6). Alternatively, women could share an initial small taste of the BEP to assuage the children’s curiosity. Family members also could intervene in support:

My child wanted to eat it, but I didn’t give it to them. When I took it with my finger, my child would say, ‘Give me some.’ My husband would then say, ‘This is for your mother,’ and the child stopped asking. (Woman, post-secondary education)

These interactions show how everyday household dynamics, including children’s food requests, could complicate women’s ability to consistently consume the supplements. They also reveal how familiy members like husbands could reinforce women’s capability to adhere.

#### Opportunity

Within the COM-B framework, opportunity is defined as the external factors outside an individual that facilitate a behaviour; these can be physical or social factors.^42^ In our study, we defined opportunity as a woman’s broader familial environment, with its attendant access to information and financial resources. Family hierarchy and decision-making played an important role in shaping women’s opportunity to adhere to supplements. Husbands and mothers-in-law especially influenced access to information, control over household resources, and distribution of food within the household.

Education appeared to play an important role in shaping participants’ uptake and consistent use of antenatal supplements. Educated women were described by CHRWs as more open to learning about the intervention and more likely to accept it. CHRWs also felt that women with an education remained more active participants in study monitoring. In one focus group, the CHRWs described how,

Many educated mothers are aware of our visit dates; I mark our going date on our calendar, so they know when we’re coming. If they have to go somewhere, they wait for us for a little while. If we’re late to call, they check in to see where we are. (FGD TBC-02, Participant 6).

In this way, women’s educational background expanded their opportunity to engage with the intervention by increasing their ability to interact with CHRWs and actively manage their own supplementation routines. While educated women were more likely to engage actively in supplementation activities, some educated family members, especially husbands, exhibited scepticism towards supplements. Differences in the generational and educational background in the household also influenced women’s opportunities for adherence. All mothers-in-law had less formal education than their daughters-in-law; nonetheless, some highly valued the BEP supplements, although for practical reasons. As one mother-in-law mentioned,

BEP keeps the body healthy. My daughter-in-law did all the housework, sweeping the yard, and everything. There was no problem. Even the day the baby was born, she was still cooking. (Family K: Mother-in-law, no education).

Conversely, some mothers-in-law had a more hesitant attitude toward nutritional supplements due to traditional beliefs and were reported to constrain opportunities to consistently adhere to nutritional supplements in pregnancy. As one of the CHRWs recounted,

Some mothers-in-law say not to eat certain foods, fearing the baby will become too fat […] when they live with their in-laws, the mothers don't get much food—no fish, meat, milk, or eggs every day (FGD TBC-07, Participant 2).

Again, these behaviours were largely reported by the CHRWs and not directly by the families themselves. Rather, families often underscored that they fed the women as usual or even emphasised that they had offered them special foods. However, lower socioeconomic status could decrease the availability of nutritious foods in the daily diet.

Socioeconomic status also influenced families’ reflections on their willingness to pay if—in the future—BEP became available for sale (the product was provided for free in the trial). For many families, significant financial barriers existed, and women’s nutrition was just one of multiple competing demands on the household budget. One participant recounted how,

Sometimes, even 1 taka isn’t available. So, how can I buy food every day? […] My family includes many people to feed. In such cases, I can’t buy or eat anything (Family F: Woman, primary education).

In general, higher SES households had more discretionary income to spend on things that were beneficial for the young mother. One high SES woman doubted her family would want to buy BEP for her at 100 taka per day, but ultimately concluded,

If it’s beneficial, I would have to buy it and eat it. Like when we go to the doctor and they say it will cost 500 or 1000 taka for a file, medicine, or injection, we immediately buy it. (Family I: Woman, junior secondary education)

Her husband, when asked if it could be purchased at 100 taka per day for 6 months in the GI, was more expansive noting,

Even if it’s 200 Taka, I can feed her. I have no financial issues. Families like mine won't face any problems. (Family I: Husband, junior secondary education)

The woman’s mother-in-law was also supportive although less enthusiastic, affirming that she would ‘allow’ the purchase at 100 taka per day for 180 days ‘Because it would be good for her health’ (Family I: Mother-in-law, no education).

These dialogues reveal how financial decision-making authority within a household, often held by the husband or mother-in-law, could shape women’s opportunity to access supplements if they required out-of-pocket expenditures. Notably, these conversations also reveal varying degrees of financial autonomy. While mothers-in-law and husbands made financial decisions in most families, in some families, women and their husbands made spending decisions independently.

When participants were asked to name maximum prices, or the price they believed BEP might cost in stores, women named higher prices than their family members. Women believed it would be sold on average at 69 taka (IQR: 20–100) per packet, while husbands named a maximum price of 68 taka (IQR: 33–88), and mothers-in-law suggested a mean price of 45 taka (IQR: 29–55). The largest variation was seen among women; only women and husbands named maximum prices that met or exceeded the actual current price per BEP packet (85 taka).

Participants provided varied rationales for their ‘best price.’ Several discussed how household dynamics can play a role in determining spending on women’s diets. As one mother-in-law shared,

Some women don’t even have the courage to ask their husbands for food. Not all men in Bengal are the same. Not all treat their wives the same way. There are some men who keep track of everything their wife needs, while there are others who don’t care about what their wife eats (Family L, Mother-in-law, no education).

Other participants offered practical examples linking the packet to something they knew and that was within their realm of decision-making agency. For example, one woman described how,

If it’s 10–15 taka, I could eat one every day. If I sell an egg, it would be 15 taka. Women usually have it. (Family I, Woman, junior secondary education)

When participants reflected on whether they could personally afford to buy one packet per day over the last 6 months of pregnancy, they increasingly emphasised the poverty of their communities. Several family members broke down during the discussion; one mother-in-law, in tears, shared,

My heart wants to feed, wants to do something, but the family just can’t. (Family M, Mother-in-law, no education)

Families also discussed rationing as a potential way to bridge their desire to provide adequate nutrition with their insufficient resources. In one family, the woman participant shared how,

If it’s 10–15 taka, I think I can eat it every day. But if it’s more than 20 taka, it would become difficult. (Family K: Woman, higher-secondary education)

Her husband and mother-in-law concurred with this view in the GI. These dialogues illustrate how gendered household power dynamics and financial constraints may limit women’s opportunity to secure additional nutrition, even when it is recognised as beneficial.

#### Motivation

COM-B conceptualises motivation as all of the cognitive processes that encourage a behaviour, many of which are unconscious.^42^ For our study, we considered motivation in the broader bio-ecological context, where it represented how different actors in the woman’s environment, including her family members, neighbours and CHRWs, affected her feelings about consumption of supplements.

For almost all participants, neighbours played an important role in motivating women’s use of antenatal supplements. Neighbours were described as ever-present and ever curious and often a source of negative motivation, leading to lower adherence to the supplements. Women described how,

Sometimes I heard what people said, sometimes I didn’t take [the MMS]. […] Some people say the child will grow big. People talk about this. That’s why I haven’t been eating properly (Family J: Woman, secondary education).

Similar messages were heard about BEP.

Conversely, in some cases, neighbours were able to play an important role in increasing positive perceptions towards the supplements. Messages from the neighbours around BEP could be positively motivating, often encouraging and reinforcing BEP as beneficial for pregnant women and their babies. These positive community messages motivated not only the pregnant women but also facilitated positive perceptions of the product among other family members.

To keep women motivated, CHRWs emphasised the need to engage their families. Given women’s many competing household responsibilities and the risk of her forgetting to take the supplement, family involvement was seen as crucial. As one CHRW noted:

Mothers have various responsibilities: cooking, the children’s studies, taking care of cows, goats and sheep, and countless other tasks. After doing all this, they might forget to take their medication. I tell the family, especially the husband and children, that it’s their duty to remind her if she forgets. (Participant 7, FGD TBC-02)

One woman recalled how the CHRW advised her husband to support her in taking the supplements, while her husband and mother-in-law also confirmed receiving messages from the CHRW about the nutritional benefits for both mother and baby.

Older children were active members of many households and were interested in their mother’s supplementation. Some CHRWs involved these children to reinforce adherence, including marking the supplement consumption calendar. As one CHRW described:

For example, a small child who is in class 5 or class 4, we remind them to tell their mother to eat this food at night or any time during the day. Just remind them. Many times, the mothers forget. They forget to put the tick. The children remind them, saying ‘Mom eats, I will put the tick.’ The children put it on almost every day. I tell them, ‘Go ahead and do it.’ They do it so nicely. They help their mothers. (Participants 1 and 3, FGD TBC-02)

In other households, children also provided verbal reminders, to make sure their mothers consumed the supplements. This was especially true during mealtimes or school breaks. One woman mentioned,

My daughter is 12. She used to remind me every day ‘Mom, eat this, it’s time.’ Sometimes before going to school, she would say, ‘Mom, it’s over there, isn’t it? (Family F: Woman, primary education,)

In addition, many CHRWs acted directly to influence and motivate women’s adherence. To combat fears about supplements leading to caesarean sections, one CHRW described how she motivated a mother to continue with supplementation, tailoring her messages to the individual concern and context,

One mother expressed concern that her baby’s weight seemed to be increasing because she was regularly taking the supplement. She questioned whether it was safe to continue and worried that it might lead to a caesarean delivery. I reassured her that if complications were to occur, they would happen regardless of the supplement. I explained that just as a paddy field yields better crops when given the right oil and fertiliser, a pregnant woman’s body also benefits from proper nutrition, which supports the healthy growth of the baby (Participant 5, FGD TBC-08)

The CHRWs also framed supplement use in terms of dietary gaps, encouraging consumption of BEP and MMS to help make up for missing nutrients,

Eat [the supplements] properly, one a day, so that no meal is missed. I see that you don’t have good access to fish, meat, milk, eggs, fruits, liver, vegetables, etc., on a daily basis. So now you should have what you can get, along with this nutritious packet, which will fulfill the rest of your nutritional needs (FGD TBC-08, Participant 2)

Additional motivational strategies included the use of tools such as calendars and pamphlets to help women track their supplement intake and conducting regular follow-ups, both in person and by phone, to track progress and troubleshoot adherence barriers. These were facilitated, according to participants, by the CHRW’s additional roles as a neighbour and a family member. Due to their presence in the community, CHRWs were able to drop in on families, answer questions, and assuage concerns.

### Discussion

The study explored the family and community dynamics influencing the uptake of and experience with the use of two antenatal nutritional supplements. Women’s capabilities, opportunities and motivation to adhere to BEP were influenced by a range of actors in their homes, in their communities and by the supplement providers called ‘Apa’ or big sister, who were the study CHRWs. Capability was enhanced when family members, including husbands and children, understood the value of the supplements and actively supported their consumption. Educational attainment and the availability of household resources played a crucial role in providing the opportunity for consistent supplement use. Motivation was shaped by various actors, including neighbours who could either support or hinder adherence through the transmission of rumours and taboos, and CHRWs who tailored adherence messages to the local context and women’s specific concerns. These findings underscore that antenatal supplement adherence is a relational behaviour embedded within overlapping household and community domains. They also reiterate the importance of a holistic approach to antenatal service delivery that engages not only the women but also their families and communities to improve antenatal supplement adherence and maternal-infant health.

#### Engaging the household and communities

Engaging men may be one approach to creating more concordance in the household around the adoption of new interventions, which has been shown to be supportive for women’s health behaviour in Bangladesh.^43^ Male engagement strategies have been shown to improve women’s adherence to health interventions through several key mechanisms. For example, when men are actively engaged, they provide practical, financial and emotional support. Their involvement can also lead to increased communication by couples and more equitable decision-making.^44 45^ Interventions that educate men through group sessions, community dialogues or peer counselling can also improve their understanding of the importance of supporting women’s health, leading to more active involvement in health-related decisions.^46^

In Bangladesh, previous nutrition programmes have incorporated similar interventions, including the direct counselling of husbands, the establishment of husband’s forums and video-based social behaviour promotions targeting men in order to engage these key household decision-makers.^11 20 24 47^ Husbands who engaged in these activities better understood maternal nutrition and their role in promoting it, and they contributed substantially to women’s adherence to antenatal supplementation.^24^ Women also appear to desire this engagement from their husbands.^48^ While husbands can be a difficult population to access, given their multiple competing economic demands and the cultural perception that health and nutrition are female spheres of influence,^24 30^ their purposeful inclusion in programme interventions can have profound impacts on women’s health and should be prioritised in Bangladesh and other contexts. Engaging men can also have more widespread societal impacts by challenging harmful gender norms that inhibit women from adhering to health interventions and services.

Surprisingly, children were also an important source of motivation for women in our study. Literature on the role of children in supporting their mothers’ participation in health interventions is limited. However, existing research highlights several potential mechanisms for better leveraging this support. Resources such as calendars or digital reminders can be designed to engage both mothers and children, promoting joint activities in health interventions.^49^ CHWs can also actively engage children in educational sessions or community health events, creating shared experiences and empowering children as advocates.^50^

Bangladesh already has several community engagement platforms such as courtyard sessions that focus on improving maternal nutrition and health, which are actively supported by both NGOs and the Ministry of Health.^10^ It has been shown that these existing structures could be strengthened and more strategically used to promote supplement use, support women’s decision-making and reduce the burden placed on individual women to justify or defend their health behaviours.^51 52^ Such platforms could help mitigate the opportunity costs associated with attending facility-based antenatal care to obtain supplements, a challenge women in the TARGET-BEP trial avoided through home delivery. These insights are directly relevant to the ‘Bangladesh National Strategy for Maternal Health (2019–2030),^10^ which calls for strengthening community-level antenatal services and improving quality of care. Integrating structured family-inclusive counselling into existing government and NGO-supported community maternal health platforms may enhanceiron-folic acid supplementation, MMS and future BEP programming under national nutrition strategies, with important implications for the expanded role of CHWs.

Indeed, CHWs in Bangladesh are influential and independent behaviour change agents, developing and adapting messages based on what they think is most likely to resonate with their clients, as demonstrated in both our study and previous work in rural Bangladesh.^53^ Working with them to document and further develop their adapted messages may be an important next step. Specific messages that may appeal to mothers-in-law, based on our findings, could include focusing on the role of supplements in keeping women strong and healthy throughout pregnancy and limiting their need for additional medical interventions. This may be especially important in future programmes: while in this study, the supplements were delivered without cost, future iterations may necessitate out-of-pocket charges or travel expenses. Paying these costs will require the husband’s support to continue women’s access to and adherence to supplements during pregnancy. This dynamic has been recognised in other ongoing supplementation programmes in Bangladesh, including the pilot of MMS in Bhola and Kurigram districts.^42^

#### Education and economic empowerment

Women’s educational status also represents an opportunity for encouraging uptake of antenatal nutrition interventions. Among our participants, the mean years of education was 8, likely due to significant investments by the Government of Bangladesh in promoting female education in recent decades.^54^ This differed strongly from the situation of mothers-in-law, who were universally uneducated; it also meant that many women respondents were more educated than their spouses. Education can help women challenge traditional gender norms, better navigate family dynamics and increase the priority of maternal and child health services.^55 56^ We saw among our participants high interest, engagement and attentiveness to the intervention, which CHRWs largely attributed to the higher educational status of young women in the study area.

Education may also help women re-examine historical sociocultural taboos affecting pregnancy diets.^5^ Rural Bangladeshi women with higher educational attainment have been seen to more effectively resist harmful food taboos^52^; these resistant behaviours were reinforced when their husbands were similarly well educated and when their families had benefited from nutritional counselling from a CHW.^52^ In our study, while mothers-in-law and older neighbours were reported to frequently suggest dietary restrictions during pregnancy or imply that supplementation might increase the risk of caesarean section, few women participants echoed these opinions. Instead, most expressed regret about having been influenced by such beliefs, indicating a recognition of their potentially harmful impact. We heard examples of women resisting restrictive mothers-in-law, although covertly, when they received support and counsel from the CHRWs. The broader advancements in women’s educational status may help underpin behaviour change interventions to help mitigate these and similar unhelpful antenatal nutrition behaviours; it may also signal the importance of engaging husbands who, like the CHRWs, could similarly reinforce women’s agency in these intrafamilial interactions.

Additionally, as more women gain access to education, they develop critical thinking skills that enable them to assert their rights and actively participate in decision-making processes within families, including those related to finances.^57 58^ This shift contributes to changing perceptions and self-perceptions of women’s roles. It has been documented that women in Bangladesh who have more education are more likely to engage in household decision-making, including financial decisions.^57 59^ Microfinance loans and other financial training appear to encourage this trajectory further.^60 61^ These shifts in household financial decision-making are already evident in our study population and offer a promising basis for future interventions to shape women’s opportunities to allocate budget towards the purchase of antenatal nutritional supplements, services and travel to obtain them.

Women’s decision-making role in the household is also known to vary based on her age, years of education and the socioeconomic status of the household.^60^ And while families in higher socioeconomic strata may have more resources available to spend on women’s health, husbands in these households may play a more dominant, less inclusive role in decision-making, regardless of women’s educational status.^62^ Thus, while education may serve as a mechanism to increase women’s opportunities to engage with antenatal health and nutrition services, education alone will be insufficient if only subgroups of women are able to benefit and broader gender norms, age norms and sociocultural beliefs remain unaddressed.

Finally, not all women will find themselves in communities or households—either in their natal or marital homes—where pregnancy nutrition will be prioritised. While women with higher educational status and greater financial autonomy may face fewer obstacles, although this is not uniformly the case,^63^ all women can benefit from support in navigating the complex systems and pressures affecting their health and nutrition decision-making. Health workers at both community and facility level can equip mothers with such strategies; again, the approaches used by the CHRWs in our study may prove useful. Similarly, programmes should consider implementing community-based interventions that actively engage not only women but broader social networks including men and other influential household members, in discussions about maternal nutrition and gender roles. By involving community members and addressing gender norms directly, such initiatives can create a more enabling environment for women to access nutrition information and services. This aligns with the WHO recommendations on ANC for a positive pregnancy experience, which call for context-specific, woman-centred care that recognises the social influences moulding health behaviours.^64^ Notably, this will be more time consuming and will require additional financial resources, although the impacts may be more substantial and sustained.

### Strengths and limitations

This study benefits from incorporating multiple perspectives, including women, their husbands, mothers-in-law and CRHWs, allowing for triangulation across household and community actors. The study also applied the COM-B framework embedded within the bioecological model to guide a theory-informed analysis. The analysis draws data from an effectiveness trial, and findings should be interpreted in the light of free supplement provision and close adherence monitoring, which may limit transferability. In addition, the longstanding presence of JiVitA in the study area may have both supported adherence and also introduced social desirability bias. Although QIs were external to the trial implementation team, participants may have still framed their responses in ways perceived as favourable to the JiVitA organisation. Additionally, interviews were conducted after completion of the trial. This might have introduced some recall bias of adherence behaviours. As with all qualitative research, findings are context-specific but may offer transferable insights for similar rural contexts, including offering crucial insights into the relational and social dynamics shaping antenatal supplement use and giving direction for designing more family-engaged maternal nutrition interventions going forward.

## Conclusion

The importance of family engagement for women’s health is well-established in both Bangladesh and globally. This study reinforces its ongoing importance for dietary and vitamin/mineral supplement adherence in rural settings within the country. It will be critical to act on these findings when designing maternal supplementation interventions, acknowledging women as embedded in multiple contexts, to increase the likelihood of future programme success. Key actions will include co-designing messages for husbands, mothers-in-law, children and neighbours in conversation with effective CHWs, such as those working in the TargetBEP trial; equipping CHWs with flexible, family-engaging counselling strategies; and complementing women’s educational gains with gender-transformative and poverty-sensitive interventions. Embedding these approaches within existing national maternal health and nutrition programmes could strengthen implementation fidelity and sustainability, particularly as Bangladesh continues expanding antenatal nutrition interventions.

## Supplementary material

10.1136/bmjopen-2025-115088 online supplemental file 1

10.1136/bmjopen-2025-115088 online supplemental file 2

## Data Availability

Data available on reasonable request due to privacy/ethical restrictions.

## References

[1] Martin SL, McCann JK, Gascoigne E (2021). Engaging family members in maternal, infant and young child nutrition activities in low- and middle-income countries: a systematic scoping review. Matern Child Nutr.

[2] Aubel J, Martin SL, Cunningham K (2021). Introduction: a family systems approach to promote maternal, child and adolescent nutrition. Matern Child Nutr.

[3] Roy S, Bailey A, van Noorloos F (2024). Understanding the barriers affecting women’s mobility in the first- and last-mile stretches in low- and middle-income countries: a systematic review. J Transp Geogr.

[4] Haque MA, Ananna RS, Hasan N (2024). Social norms and maternal health information-seeking behavior among adolescent girls: a qualitative study in a slum of Bangladesh. PLoS ONE.

[5] Shannon K, Mahmud Z, Asfia A (2008). The social and environmental factors underlying maternal malnutrition in rural Bangladesh: implications for reproductive health and nutrition programs. Health Care Women Int.

[6] Harris-Fry HA, Paudel P, Shrestha N (2018). Status and determinants of intra-household food allocation in rural Nepal. Eur J Clin Nutr.

[7] World Bank (2016). Overview on bangladesh’s safety net program.

[8] Benova L, Tunçalp Ö, Moran AC (2018). Not just a number: examining coverage and content of antenatal care in low-income and middle-income countries. BMJ Glob Health.

[9] Training NI of PR and, Division ME and FW, Welfare M of H and F, ICF (2024). Bangladesh Demographic and Health Survey 2022: Final Report. https://dhsprogram.com/publications/publication-fr386-dhs-final-reports.cfm.

[10] Government of the People’s Republic of Bangladesh: Ministry of Health and Family Welfare (2019). Bangladesh national strategy for maternal health: 2019-2030. https://dgnm.portal.gov.bd/sites/default/files/files/dgnm.portal.gov.bd/page/18c15f9c_9267_44a7_ad2b_65affc9d43b3/2021-06-24-11-27-702ae9eea176d87572b7dbbf566e9262.pdf.

[11] Flax VL, Bose S, Escobar-DeMarco J (2025). Changing maternal, infant and young child nutrition practices through social and behaviour change interventions implemented at scale: Lessons learned from Alive & Thrive. Matern Child Nutr.

[12] Sanghvi T, Haque R, Roy S (2016). Achieving behaviour change at scale: alive & thrive’s infant and young child feeding programme in Bangladesh. Matern Child Nutr.

[13] Abdulloeva S, Bhanot A, Khan MA (2025). Centering community-based maternal and child nutrition services in Bangladesh’s rural primary healthcare: what has potential to scale. Front Public Health.

[14] Furstenberg FF (2019). Family change in global perspective: how and why family systems change. Fam Relat.

[15] Sung S, Smyth L (2022). Gendered families: states and societies in transition. Contemp Soc Sci.

[16] Kibria GMA, Albrecht J, Lane W (2024). Prevalence, trends, and factors associated with maternal autonomy regarding healthcare, finances, and mobility in Bangladesh: analysis of demographic and health surveys 1999-2018. *PLOS Glob Public Health*.

[17] Haider MR, Qureshi ZP, Khan MM (2017). Effects of women’s autonomy on maternal healthcare utilization in Bangladesh: evidence from a national survey. Sexual & Reproductive Healthcare.

[18] Kabeer N (2011). Between affiliation and autonomy: navigating pathways of women’s empowerment and gender justice in rural Bangladesh. Dev Change.

[19] Hoddinott J, Ahmed I, Ahmed A (2017). Behavior change communication activities improve infant and young child nutrition knowledge and practice of neighboring non-participants in a cluster-randomized trial in rural Bangladesh. PLoS One.

[20] Nguyen PH, Frongillo EA, Kim SS (2019). Information diffusion and social norms are associated with infant and young child feeding practices in Bangladesh. J Nutr.

[21] Smith ER, Gomes F, Adu-Afarwuah S (2025). Contribution of maternal adherence to the effect of multiple micronutrient supplementation dring pregnancy: a systematic review and individual participant data meta-analysis. Adv Nutr.

[22] Kang Y, Workneh F, Yibeltal K (2024). Effect of a prenatal balanced energy-protein (BEP) supplement on gestational weight gain in rural Ethiopia. Current Developments in Nutrition.

[23] Zavala E, Rahman A, Kalbarczyk A (2024). Acceptability of a balanced energy protein (BEP) supplement for pregnant women in Bangladesh. Matern Child Nutr.

[24] Nguyen PH, Frongillo EA, Sanghvi T (2018). Engagement of husbands in a maternal nutrition program substantially contributed to greater intake of micronutrient supplements and dietary diversity during pregnancy: results of a cluster-randomized program evaluation in Bangladesh. J Nutr.

[25] Kraemer K, Beesabathuni K, Askari S (2023). Knowledge, attitudes and practices of pregnant women and healthcare providers in Bangladesh regarding multivitamin supplements during pregnancy. Healthcare (Basel).

[26] Woods SB, Bridges K, Carpenter EN (2020). The critical need to recognize that families matter for adult health: a systematic review of the literature. Fam Process.

[27] Zavala E, Mohan D, Ali H (2024). Targeting strategies for balanced energy and protein (BEP) supplementation in pregnancy: study protocol for the TARGET-BEP cluster-randomized controlled trial in rural Bangladesh. Trials.

[28] Morgan DL, Ataie J, Carder P (2013). Introducing dyadic interviews as a method for collecting qualitative data. Qual Health Res.

[29] Labrique AB, Christian P, Klemm RDW (2011). A cluster-randomized, placebo-controlled, maternal vitamin A or beta-carotene supplementation trial in Bangladesh: design and methods. Trials.

[30] Khaled N, Kalbarczyk A, Zavala E (2024). A formative study of the sociocultural influences on dietary behaviours during pregnancy in rural Bangladesh. Matern Child Nutr.

[31] Gunnsteinsson S, Labrique AB, West KP (2010). Constructing indices of rural living standards in Northwestern Bangladesh. J Health Popul Nutr.

[32] Hennink M, Kaiser BN (2022). Sample sizes for saturation in qualitative research: a systematic review of empirical tests. Soc Sci Med.

[33] Proctor E, Silmere H, Raghavan R (2011). Outcomes for implementation research: conceptual distinctions, measurement challenges, and research agenda. Adm Policy Ment Health.

[34] Bingham AJ (2023). From Data Management to Actionable Findings: A Five-Phase Process of Qualitative Data Analysis. Int J Qual Methods.

[35] Flick U (1992). Triangulation Revisited: Strategy of Validation or Alternative?. J Theory Soc Behav.

[36] Peh KQE, Kwan YH, Goh H (2021). An Adaptable Framework for Factors Contributing to Medication Adherence: Results from a Systematic Review of 102 Conceptual Frameworks. J Gen Intern Med.

[37] O’Brien BC, Harris IB, Beckman TJ (2014). Standards for reporting qualitative research: a synthesis of recommendations. Acad Med J Assoc Am Med Coll.

[38] Michie S, van Stralen MM, West R (2011). The behaviour change wheel: A new method for characterising and designing behaviour change interventions. Implementation Sci.

[39] Brofenbrenner U, Morris PA (2006). Handbook of child psychology, theoretical models of human development.

[40] Willmott TJ, Pang B, Rundle-Thiele S (2021). Capability, opportunity, and motivation: an across contexts empirical examination of the COM-B model. BMC Public Health.

[41] Gregg K, Rickert NP, Leckie A (2024). Following the family: applying bioecological theory to strategies learned from a family-school-community partnership. Sch Community J.

[42] Healthy Mothers Healthy Babies Consortium (2021). Formative Research for the Introduction of Multiple Micronutrient Supplements in Bangladesh. https://hmhb.micronutrientforum.org/knowledge-hub/formative-research-for-the-introduction-of-multiple-micronutrient-supplements-in-bangladesh/.

[43] Uddin J, Hossin MZ, Pulok MH (2017). Couple’s concordance and discordance in household decision-making and married women’s use of modern contraceptives in Bangladesh. BMC Womens Health.

[44] Gottert A, Pulerwitz J, Weiner R (2025). Systematic review of reviews on interventions to engage men and boys as clients, partners and agents of change for improved sexual and reproductive health and rights. BMJ Open.

[45] Tokhi M, Comrie-Thomson L, Davis J (2018). Involving men to improve maternal and newborn health: a systematic review of the effectiveness of interventions. PLoS One.

[46] Nabia S, Betron M, Arlotti-Parish E (2025). Strategies for men’s engagement and its effectiveness in improving child health and immunization-a rapid review. Front Public Health.

[47] Quisumbing A, Ahmed A, Hoddinott J (2021). Designing for empowerment impact in agricultural development projects: experimental evidence from the agriculture, nutrition, and gender linkages (ANGeL) project in Bangladesh. World Dev.

[48] Rahman AE, Perkins J, Salam SS (2020). What do women want? An analysis of preferences of women, involvement of men, and decision-making in maternal and newborn health care in rural Bangladesh. BMC Pregnancy Childbirth.

[49] Gilano G, Dekker A, Fijten R (2024). The role of mHealth intervention to improve maternal and child health: a provider-based qualitative study in southern Ethiopia. PLoS One.

[50] Willis E, Godbold R (2024). Children’s complex health: maternal experiences of care and decision making. J Child Health Care.

[51] Ministry of health and family welfare (2016). Comprehensive social and behaviour change communication strategy. https://www.bangladesh-ccp.org/assets/images/resources/completion/Comprehensive%20SBCC%20Strategy-3-.pdf.

[52] Rayna SE, Khan FA, Samin S (2025). Motivators for adherence and drivers of taboo-breaking behaviour regarding food taboos among rural pregnant women in Bangladesh: findings from formative research. Matern Child Nutr.

[53] Wable Grandner G, Rasmussen KM, Dickin KL (2022). Storytelling for persuasion: insights from community health workers on how they engage family members to improve adoption of recommended maternal nutrition and breastfeeding behaviours in rural Bangladesh. Matern Child Nutr.

[54] Khandker S, Pitt M, Fuwa N Subsidy to promote girls’ secondary education: the female stipend program in bangladesh.

[55] Pike V, Kaplan Ramage A, Bhardwaj A (2021). Family influences on health and nutrition practices of pregnant adolescents in Bangladesh. Matern Child Nutr.

[56] Pratley P (2016). Associations between quantitative measures of women’s empowerment and access to care and health status for mothers and their children: a systematic review of evidence from the developing world. Soc Sci Med.

[57] Chanda SK, Howlader H, Nahar N (2012). Educational status of the married women and their participation at household decision making in rural Bangladesh. Int J Adv Res Amp Technol.

[58] Stromquist NP (2015). Women’s empowerment and education: linking knowledge to transformative action. Eur J Educ.

[59] Kirkwood EK, Raihana S, Alam NA (2024). Women’s participation in decision‐making: analysis of Bangladesh demographic and health survey data 2017–2018. J of Intl Development.

[60] Mahmud S, Shah NM, Becker S (2012). Measurement of Women’s Empowerment in Rural Bangladesh. World Dev.

[61] Kabeer N (2001). Conflicts Over Credit: Re-Evaluating the Empowerment Potential of Loans to Women in Rural Bangladesh. World Dev.

[62] Goetz AM, Gupta RS (1996). Who takes the credit? gender, power, and control over loan use in rural credit programs in Bangladesh. World Dev.

[63] Shourove JH, Meem FC, Rahman M (2023). Is women’s household decision-making autonomy associated with their higher dietary diversity in Bangladesh? evidence from nationally representative survey. *PLOS Glob Public Health*.

[64] WHO recommendations on antenatal care for a positive pregnancy experience. https://www.who.int/publications/i/item/9789241549912.

