# Supplementary material for: Exploring the interplay of family dynamics and pregnancy supplement adherence among married women of reproductive age: a qualitative study from rural Bangladesh
Source: BMJ Open. 2026 May 12;16(5):e115088. doi: 10.1136/bmjopen-2025-115088 (PMC13182426; doi:10.1136/bmjopen-2025-115088)
Supplement: online supplemental file 1 [file bmjopen-16-5-s001.docx]

**S1: Supplementary Materials**

**Codebook Implementation Outcomes Study**

[**1. Supplements Family**](#_jby05rq2034t)

[**2. Acceptability Family**](#_uq1rkzab3p29)

[**3. Adherence Family**](#_owablpjad24g)

[Promoters sub-family](#_2q9mk16y33jl)

[Obstacles sub-family](#_gj6ms8bo1o90)

[Could go either way](#_mm2a25d64qcq)

[**4. Benefits Family**](#_tjx07ua129wu)

[**5. Other Family**](#_7ulxolde6pc)

| 1. Supplements Family | | To keep track of which supplement is being described in a particular coded chunk | | | |
| --- | --- | --- | --- | --- | --- |
| Code Name | Brief Description | Full Definition | When to Use | When Not to Use | Example Passages |
| mms | MMS | - | This is always a double code. Apply this code whenever interviewees are talking about MMS. | Alone. | - |
| bep | BEP | - | This is always a double code. Apply this code whenever interviewees are talking about BEP | Alone. | - |
| poly | Multiple medicines | Taking medicines in addition to JiVitA supplements | Women discuss using medicines or supplements in addition to BEP and MMS | When women are talking about MMS only. | “Yes, I went to the doctor. They gave me calcium tablets, which I took, and later they gave me iron tablets.” |

| 2. Acceptability Family | | The degree to which the product is agreeable, palatable, or satisfactory | | | |
| --- | --- | --- | --- | --- | --- |
| Code Name | Brief Description | Full Definition | When to Use | When Not to Use | Example Passages |
| senses | Sensory components | Taste, texture, smell, palatability | When women discuss aspects of the product that they like/dislike | When talking about remembering to take the product (adherence) or suitability of the product (appropriateness) | “The green one tasted better to me, and the red one caused more gas. It also had a stronger smell. I couldn’t tolerate the smell at that time because I was pregnant.” |
| side effects | Unintended side effects linked to the product that occur during pregnancy | These can be actual or perceived negative side effects that decrease acceptability | When women talk about how the product:   - Made them feel sick or nauseous - Caused gas or headaches - Had other side effects | When discussing a birth outcome or something that happened after delivery | “Then they said I should take both. I said that the non-flavored one made my head spin. They told me that it would get better over time, and gradually it did. Then I ate both.” |
| outcome | Product is perceived as tied to a certain birth outcome | Describes how interviewees think the product influenced (or not) the birth outcome | When women discuss:   - C-sections - Birth abnormalities - How the baby looked - How much baby weighed - Baby’s intelligence | When discussing a side effect that occurred during the pregnancy | “My sister in law said, “You eat the nutritional packet; why is your baby like this?” I said it’s the nutritional packet. Then I went to the doctor, and he said this is not due to any food. It could be hereditary, but I don’t know why it happened. No one in our family has this issue, and neither does his father’s family. It doesn’t depend on the nutritional packet.” |
| trust | Past (positive) experiences with JiVitA | Past experiences and trust of JiVitA make the products more acceptable | This can include:   - Comparisons with eg Mother Horlicks and FullCare - Pregavita is known and therefore trusted - Reasons women accept BEP easily |  | “When the pushti packet comes, they say, "You have been giving tablets for so long, now you are giving a pushti packet." Then we say that you have received tablets and you have eaten them too. Now, to ensure a mother’s good health and well-being, if this pushti packet can be beneficial, then we are giving it.” |

| 3. Adherence Family | | Whether and why/not products were consumed at the full dosage, every day, with no sharing | | | |
| --- | --- | --- | --- | --- | --- |
| Code Name | **Brief Description** | **Full Definition** | **When to Use** | **When Not to Use** | **Example Passages** |
| strategy | Adherence strategies | Different ways women remembered to consume the product | About actions taken:   - Preparing/eating product in different ways - Where she placed it in her home - How she kept other family members from eating it - When she ate it | When not an action  When it is something the CHRW did | “I managed to eat it with milk, and that’s how I ate it.” |
| Promoters sub-family | | **Ways that outside messages, counseling, etc., positively influenced women’s adherence** | | | |
| value | Perceived value of product | The product is something that is valuable and this makes it more acceptable | Use when focused on reasons women liked/did not like the product based upon its perceived value   - contains so many vitamins - was given especially for me/for pregnant women - comparisons between past/current pregnancy - comparisons with other products - rationale for costs - it came from overseas/ was given by gov’t | When talking about benefits - benefits are actual positive things that happen as a result of taking the product  Do not code generic statements:   - It was nutritious - it was given for my benefit - I benefited | “We also give them a pamphlet that lists 15 vitamins and minerals in the supplements. This makes the mothers happy, knowing they will receive something beneficial during their pregnancy.” |
| chrw | Advice, instructions, push-back from CHRW | CHRW gives advice or takes actions that help women consume the product | When CHRWs:   - give advice on preparing the product - counsel on nutrition/benefits - describe why some women need BEP - visit/collect packets | If the action is something the woman developed or decided herself | Some mothers ask, "Why don’t you give us fruits like apples, oranges, or tangerines? That would be good food." I explain, "If we gave you fruits, you might give them to your husband or child and not eat them yourself. This supplement is made from similar nutrients. Take it." |
| other_promoter | Promoters not described above | Another promoter not described above | When there is a promoter that doesn’t fit into the groups above. | N/A | - |
| Obstacles sub-family | | **Ways that outside messages, counseling, etc., positively influenced women’s adherence** | | | |
| share | Sharing | Women give BEP or MMS to others. | When others ask for or receive some of the MMS or BEP that has been given to the mothers - can be a tiny taste or several packets. | When talking about family member reminders to eat - that is a promoter and a positive thing. | “My niece and nephew live next door. They have gone to school now. They always want to eat it. Every day they would ask, “chachi amma/aunty, let’s eat.” When I ate it, they ate it too.” |
| homeop | Homeopathic doctors or medicines | Interference from homeopathy or other complementary medicine regimes. | When women are advised to stop taking MMS or BEP by a homeopathic medical provider/due to homeopathic medicines | When the doctor gives positive advice and encourages women to consume - this is in the promoter family, “doctor” | “In the village, there is a notion about using homeopathy to ease childbirth. The doctor told her not to eat that food.She showed the tablets to him.he was a homeopathic doctor.” |
| other_obstacle | Obstacles not described above | Another obstacle not described above | When there is an obstacle that doesn’t fit into the groups above.   - Woman forgets | N/A | - |
| Could go either way | | **Impact varies** | | | |
| neighbor | Community talk | Neighbors opinions and ideas influence women’s adherence | When neighbors say things about the supplements, e.g.:   - causing C-sections - being unnecessary - causing birth defects - ask for sharing - remind mother to eat | When the individual is a family member or CHRW | “ It would have been better to have it (MMS), but I did not do so because of what neighbors were saying. It (MMS) makes the first baby fat, they said.” |
| doctor | Feedback from the doctor / encounter with the doctor | Doctors influence (or do not) women’s antenatal nutrition | Women or family discuss medical feedback on antenatal nutrition and/or the role of supplements. Can also be: we do not discuss nutrition with doctors. | When discussing homeopathy | “I had a quick pregnancy, and for that issue, I went there, and my husband showed him whether this tablet could be taken. Then the doctor said it could be taken.” |
| family | Family affects women’s eating (or not) | Family members (MIL, Husband, kids) influence women’s adherence. | When women, CHRWs, or family members describe how the family supported women’s adherence (or discouraged) | Can be co-coded with sharing - but sharing is an obstacle. | “I didn’t want to eat, but my mother-in-law would scold me, saying it’s good to eat. My husband also insisted, everyone did.” |
| types | Types of women who adhere | Characteristics of women who are more or less adherent to supplementation | When CHRWs discuss:   - stubbornness - education - generational differences | When CHRWs are discussing the counseling messages they gave -- this should be in the promote/ “CHRW” family. | “At first, it was quite difficult to get mothers to take the tablets, but now that’s not the case; today’s mothers are much more educated.”. |

| 4. Benefits Family | | The ways in which the products work in the body | | | |
| --- | --- | --- | --- | --- | --- |
| Code Name | **Brief Description** | **Full Definition** | **When to Use** | **When Not to Use** | **Example Passages** |
| gaps | Supplements are for filling gaps or deficiencies | The supplement aids the mother in filling in deficiencies in her nutritional or health status. | Use when discussing why the woman takes the supplement or how the supplement works. | Distinguished from “strong” by the fact that strong increases the body’s capabilities, whereas “gaps” is about bringing the mother back up to some baseline. | “Nutrition is nutrition; it fulfills our body’s deficiencies.”  “We can't always eat nutritious food. We can't have milk and eggs every day. This pushti packet helps to fulfill the deficiencies in the body.” |
| prevent | Supplements can prevent negative (health) consequences | The supplement has a preventive aspect– preventing problems for either the mother’s or baby’s health | Use when discussing why the woman takes the supplement or how the supplement works. | Distinguished from “gaps” and “strong” because it focuses on prevention itself not on the mechanism of action in the woman’s body. | “It also reduces illness, and they do not get sick. It is a kind of nutrition. Eating it increases the chances of normal delivery.” |
| cure | Supplements can resolve health issues | The supplement makes a health condition go away. | Women have been having negative side effects in pregnancy and the supplements make them go away. | Distinguished from “strong” as "strong” is about maintaining strength and is linked to activity; distinguished from “gaps” because gaps is about making up for a deficiency. | “My body was weak. I fainted from dizziness. But after starting to eat this, I had no issues after 5 months.” |
| strong | Supplements keep women healthy, energetic, and capable | The supplement aids the mother in staying healthy and maintaining strength and energy for work throughout the pregnancy | Use when discussing why the woman takes the supplement or how the supplement works.   - having energy - being able to work - having increased appetite - staying healthy | Distinguished from “gaps” because “strong” focuses on increasing the body’s capabilities rather than restoring body to baseline; distinguished from “prevent” because it focuses on the mechanism by which the body may evade illness. | “The benefits for pregnant mothers include that their body remains healthy all the time; eating this keeps them energized.”  “While the nutritious food was less, after eating this pushti packet, I realized that my body was benefiting from it. I felt that my body was more active because of this nutrition." |

| 5. Other Family | | Remaining implementation outcomes constructs | | | |
| --- | --- | --- | --- | --- | --- |
| Code Name | **Brief Description** | **Full Definition** | **When to Use** | **When Not to Use** | **Example Passages** |
| access | How supplementation needs to be available to other women in the community | The supplement is something that must be accessible to all kinds of women and should be formulated and/or priced accordingly | When discussing aspects of acceptability, cost, or targeting and the interviewee discusses the needs of women in the community more broadly | Different from appropriateness, as approp means they think it is right or suitable to use it, whereas access means they can use it. | “It would be better for everyone if the price were lower”  “I don’t eat it alone, it’s for everyone.” |
| target_a | Fittingness, suitability of targeting | The perceived fit, relevance, or compatibility of **targeting** for the setting or the recipient; and/or perceived fit of targeting to address maternal nutrition problems. | When discussing what kinds of women could benefit from BEP and/or what others in the community have said about BEP consumption | Different from “accept” in that acceptability is about liking the product. “Approp” is about targeting and suitability of **targeting**, | “Around me, everyone gets pushti. Those whose names are on Jivita's list, they get it, but no one else does.” |
| cost | Affordability | The ability of a household to afford maternal nutrition (either supplements or food). | When describing the impact of costs on the family budget, where funds would come from, and whether/why a price point would be affordable | N/A | “Since it has more nutrients, I think the price could be higher.” |
| religion | Religion or fate | The outcome is out of human hands | Only use as a double code -- secondary to another issue (i.e., outcome + religion) | When it is a simple exclamation (Oh, God!) | “Allah created everyone differently, right? What’s the connection between fat and the food? You’re short, I’m short, she’s tall, does that mean she has more nutrition?” |
| program | Programmatic considerations | Aspects of the program that could be changed or improved or that were particularly successful | Use when CHRWs/participants are describing how the program affected them.   - equipment, bikes, transport - eligibility criteria - adherence - affordability - workload | When describing counseling messages CHRWs gave women -- this should be in the promote/ “CHRW” family.  When discussing things that are very specific to the research trial setting: the different forms, the consent process - these should not be coded. | “Participant b: I need to take 150 packets. There’s a target, as we have to feed five mothers based on the schedule. Sometimes, I might need to take some food in the front and some in the back of the bicycle.  Participant e: I tell that apa, “You take it halfway apa.” then I take it back from her after halfway.” |
| substitute | Diet/substitution | How the supplements did/did not change the typical diet | - Perceived as a snack - Or as a meal (replacement for food) - If specifically talking about deficiency of certain foods | Potential to double code with “gaps” | “Interviewer: When you were eating this, did your regular diet change in any way?  Participant: No.” |
