# Supplementary material for: Exploring the interplay of family dynamics and pregnancy supplement adherence among married women of reproductive age: a qualitative study from rural Bangladesh
Source: BMJ Open. 2026 May 12;16(5):e115088. doi: 10.1136/bmjopen-2025-115088 (PMC13182426; doi:10.1136/bmjopen-2025-115088)
Supplement: Supplementary file 3 [file bmjopen-2025-115088.reviewer_comments.pdf]

PEER REVIEW HISTORY

BMJ Open publishes all reviews undertaken for accepted manuscripts. Reviewers are asked to complete a checklist review form and are provided with free text boxes to elaborate on their assessment. These free text comments are reproduced below.

ARTICLE DETAILS

Title (Provisional)

Exploring The Interplay of Family Dynamics and Pregnancy Supplement Adherence Among Married Women of Reproductive Age: A Qualitative Study from Rural Bangladesh

Authors

Mondal, Pragna Paramita; de Boer, Mary; Rahman, Atiya; Khaled, Nazrana; Zavala, Eleonor; Haque, Rezwanul; Ali, Hasmot; Siddiqua, Towfida Jahan; Afsana, Kaosar; Christian, Parul; Lyman, Andrew Thorne; Kalbarcyzk, Anna

---

VERSION 1 - REVIEW

**Reviewer**	**1**
**Name**	**Marian, Marian**
**Affiliation**	**University of California San Diego**
**Date**	**06-Jan-2026**
**COI**	**None**

Thank you for the opportunity to review this article! This is a very well-written article. The findings align with the journal's aims. The topic and scope of this research is both interesting and timely. I think this manuscript will be of particular interest to scholars and public health professionals focused on maternal health and supplement adherence. The focus on family dynamics represents a novel contribution to the literature. The manuscript needs minor work before it would be ready for publication in my view.

These are my suggestions:

Title:

- I suggest including the geographical location as part of the title.

Abstract:

-Remember to include what the acronym stands for the first time you use it, specifically for COM-B.

-I recommend adding sample size information to the participants or results sub-section.

Introduction:

-Page 3 lines 27-28, would you expand on what type of actors beyond the immediate household influence on health and nutrition behaviors.

-I suggest including some background information about maternal nutrition interventions in Bangladesh. Is there literature on maternal MMS/BEP adherence, behavior, or knowledge?

Methodology:

-Page 4 line 14, please specify what the four arms of the TargetBEP trial are.

-Is there a difference between group discussions and focus group discussions? If not, I suggest only using the terms FGDs (for 3 or more participants) and dyadic interviews (for husband and mother-in-law pairs).

Discussion:

I know that you included the strengths and limitations of this study at the beginning of the manuscript, but I also suggest closing your discussion section with this or with a call to the strengths or limitations.

Figures and Tables:

-For table 1, I suggest using the traditional table with borders that separate the variables rather than the hyphen next to the variable.

-I do not think that table 2 is necessary. You provided basic information on the results section, and the BEP prices are not part of the main objectives of this paper.

Other:

-Rather than including your manual of operations, I suggest you include your interview guides and an annex of additional quotes to support your themes.

Reviewer 2

Name Aji, Arif Sabta

Affiliation National Research and Innovation Agency Republic of Indonesia, Research Center for Biomedical

Date 19-Jan-2026

COI None

This manuscript presents a well-designed qualitative study exploring how family dynamics, community actors, and social networks influence adherence to pregnancy nutritional supplements in rural Bangladesh. The topic is highly relevant to maternal and public health in LMICs and aligns well with the scope of BMJ Open. The use of the COM-B framework

provides a clear analytical structure, and the inclusion of multiple perspectives (women, husbands, mothers-in-law, and community health research workers) is a notable strength.

1. Please consider strengthening the final paragraph of the Introduction and the opening of the Discussion to clearly state what this study adds beyond existing qualitative and mixed-methods literature on antenatal supplementation.

2. The Discussion would benefit from a more explicit synthesis of how these domains interact (e.g., how family support enhances both capability and motivation, or how gender and generational hierarchies shape opportunity).

3. Clarifying these interconnections would strengthen the theoretical contribution of the study.

4. The authors may wish to more explicitly interpret how specific family dynamics (e.g., authority of mothers-in-law, spousal support) function as mechanisms influencing adherence, rather than primarily illustrating these dynamics through examples.

5. Please briefly describe the positionality of the research team, particularly in relation to the TARGET-BEP trial and the implementing organization.

6. Additionally, a short justification of sample adequacy and discussion of transferability (rather than generalisability) would be appropriate, in line with qualitative research standards.

7. The limitations section could be strengthened by briefly reflecting on how these factors may have shaped participant perceptions, behaviours, and reported adherence, and how this should be considered when interpreting the findings.

8. The manuscript could be further strengthened by explicitly linking these recommendations to existing WHO antenatal care guidance or national nutrition programming in Bangladesh, highlighting how the findings can inform policy and implementation.

VERSION 1 - AUTHOR RESPONSE

Reviewer: 1
Dr. Marian Marian, University of California San Diego
Comments to the Author:

Thank you for the opportunity to review this article! This is a very well-written article. The findings align with the journal's aims. The topic and scope of this research is both interesting and timely. I think this manuscript will be of particular interest to scholars and public health professionals focused on maternal health and supplement adherence. The focus on family dynamics represents a novel contribution to the literature. The manuscript needs minor work before it would be ready for publication in my view.

These are my suggestions:

Title:
- I suggest including the geographical location as part of the title.

Response: Thank you for this helpful suggestion. We have revised the title to include the geographical location of the study. The updated title now reads: *"Exploring the Interplay of Family Dynamics and Pregnancy Supplement Adherence Among Married Women of Reproductive Age: A Qualitative Study from Rural Bangladesh."*

Abstract:
-Remember to include what the acronym stands for the first time you use it, specifically for COM-B
Response: Thank you for these helpful suggestions. We have revised the abstract to spell out the COM-B framework in full at first mention as the Capability, Opportunity, Motivation-Behavior (COM-B) framework**.**

-I recommend adding sample size information to the participants or results sub-section.
Response: We have added the sample size information to the Participants section of the abstract

Introduction:
-Page 3 lines 27-28, would you expand on what type of actors beyond the immediate household influence on health and nutrition behaviors.
Response: Thank you for these constructive suggestions. In response, we have expanded the Introduction to more clearly specify the types of actors beyond the immediate household who influence women's health and nutrition behaviors. We now explicitly reference neighbors, community leaders, and community health workers as important social actors shaping maternal nutrition practices.

-I suggest including some background information about maternal nutrition interventions in Bangladesh. Is there literature on maternal MMS/BEP adherence, behavior, or knowledge?
Response: Thank you for this helpful suggestion. We have added a brief sentence in the Introduction (maternal nutrition interventions paragraph) summarizing existing evidence from Bangladesh on IFA and MMS adherence and the influence of social and household factors.

Methodology:
-Page 4 line 14, please specify what the four arms of the TargetBEP trial are.
Response: Thank you for this suggestion. We have revised the Methodology section to clearly specify the four arms of the TargetBEP trial and to briefly describe the supplementation strategy

-Is there a difference between group discussions and focus group discussions? If not, I suggest only using the terms FGDs (for 3 or more participants) and dyadic interviews (for husband and mother-in-law pairs).
Response:. Yes, the group discussions and focus group discussions are not the same. In our original protocol, the interview with husbands and mothers-in-law these were described as group interviews. However, we agree that they were conducted in a dyadic format, as each session included two participants (husband and mother-in-law pairs). To improve clarity while remaining consistent with the protocol terminology, we have revised the manuscript to describe them as "group interviews conducted in a dyadic format" under the study design section

Discussion:
I know that you included the strengths and limitations of this study at the beginning of the manuscript, but I also suggest closing your discussion section with this or with a call to the strengths or limitations.
Response: Thank you for this thoughtful suggestion. We have revised the discussion section to conclude with a concise paragraph that reiterates the key strengths and limitations of the study.

Figures and Tables:
-For table 1, I suggest using the traditional table with borders that separate the variables rather than the hyphen next to the variable.
Response: Thank you for these helpful suggestions regarding the tables. We have reformatted Table 1 to use a traditional table layout with borders separating variables and categories

-I do not think that table 2 is necessary. You provided basic information on the results section, and the BEP prices are not part of the main objectives of this paper.
Response: We agree that Table 2 was not central to the main objectives of the paper. As suggested, we have removed Table 2 from the manuscript

Other:
-Rather than including your manual of operations, I suggest you include your interview guides and an annex of additional quotes to support your themes.
Response: Thank you for this helpful suggestion. In line with your recommendation, we have included the interview guides used for the in-depth interviews, group interviews, and focus group discussions as supplementary materials to increase transparency regarding the data collection process. Regarding the inclusion of additional quotations in an annex, we did not add further quotations because the Results section already includes multiple illustrative quotes for each theme, which we believe adequately represent the range of participant perspectives captured in the data.

Reviewer: 2
Dr. Arif Sabta Aji, National Research and Innovation Agency Republic of Indonesia
Comments to the Author:
This manuscript presents a well-designed qualitative study exploring how family dynamics, community actors, and social networks influence adherence to pregnancy nutritional supplements in rural Bangladesh. The topic is highly relevant to maternal and public health in LMICs and aligns well with the scope of BMJ Open. The use of the COM-B framework provides a clear analytical structure, and the inclusion of multiple perspectives (women, husbands, mothers-in-law, and community health research workers) is a notable strength.

1. Please consider strengthening the final paragraph of the Introduction and the opening of the Discussion to clearly state what this study adds beyond existing qualitative and mixed-methods literature on antenatal supplementation.
Response: Thank you for this helpful suggestion. In response, we have revised the final paragraph of the Introduction to more clearly articulate the study's objective and contribution. We now explicitly state that the paper examines how household power dynamics within interconnected family and community systems shape antenatal supplement adherence, drawing on data embedded within an effectiveness trial.

2. The Discussion would benefit from a more explicit synthesis of how these domains interact (e.g., how family support enhances both capability and motivation, or how gender and generational hierarchies shape opportunity).
Response: Thank you for this helpful suggestion. We have added a short integrative paragraph in the Discussion section, immediately following the summary paragraph on capability, opportunity, and motivation. This addition explicitly synthesizes how these domains interact within family and community systems, highlighting how family support, generational hierarchies, and CHRW engagement operate across multiple behavioral domains simultaneously.

3. Clarifying these interconnections would strengthen the theoretical contribution of the study.
Response: Thank you for this thoughtful comment. We have clarified the interconnections between the COM-B domains and the embedded bio-ecological systems in both methods and the discussion sections
.
4. The authors may wish to more explicitly interpret how specific family dynamics (e.g., authority of
mothers-in-law, spousal support) function as mechanisms influencing adherence, rather than primarily illustrating these dynamics through examples.
Response: Thank you for this helpful suggestion. We have revised the Results section to more explicitly interpret how family dynamics function as mechanisms influencing women's adherence to antenatal supplementation. In particular, we have added interpretive statements clarifying the roles of husbands, mothers-in-law, natal families, and children in shaping women's capability, opportunity, and motivation within the COM-B framework

5. Please briefly describe the positionality of the research team, particularly in relation to the TARGET-BEP trial and the implementing organization.
Response: Thank you for this important comment. In response, we have strengthened the Reflexivity Statement to more explicitly describe the positionality of the research team in relation to the TARGET-BEP trial and the implementing organization. We now clarify which team members were involved in trial design and implementation, which researchers were external to day-to-day trial delivery, and how this positioning may have influenced data collection, interpretation, and participant responses. We also describe the steps taken to mitigate potential bias, including the use of qualitative interviewers who were not part of the trial implementation team.

6. Additionally, a short justification of sample adequacy and discussion of transferability (rather than generalizability) would be appropriate, in line with qualitative research standards.
Response: Thank you for this helpful suggestion. We have added a brief justification of sample adequacy in the Methods section. Specifically, we clarified that data collection continued until thematic saturation was reached and included supporting evidence from empirical research indicating that 9–17 interviews or 4–8 focus group discussions are typically sufficient to achieve thematic saturation in qualitative studies. Based on this evidence, we note that the sample size used in our study was considered adequate. In addition, we expanded the Discussion section to address transferability rather than generalizability, noting that while the findings are context-specific to rural Bangladesh and the TARGET-BEP trial setting, they provide insights into family and community dynamics shaping antenatal supplement adherence that may be relevant to similar sociocultural contexts.

7. The limitations section could be strengthened by briefly reflecting on how these factors may have shaped participant perceptions, behaviors, and reported adherence, and how this should be considered when interpreting the findings.

Response: Thank you for this helpful suggestion. In response, we have strengthened the Limitations section in the Discussion to explicitly reflect how the trial context (free home delivery of supplements and close adherence monitoring), JiVitA's longstanding presence in the study area, and the timing of post-trial interviews may have shaped participant perceptions, behaviors, and reported adherence. We have also clarified that findings should be interpreted within this supportive trial environment

8. The manuscript could be further strengthened by explicitly linking these recommendations to existing WHO antenatal care guidance or national nutrition programming in Bangladesh, highlighting how the findings can inform policy and implementation.

Response: Thank you for this helpful suggestion. We have revised the Discussion section (subsections: "Engaging the household and communities" and "Education and economic empowerment") to explicitly link our findings to the WHO Recommendations on Antenatal Care (2016) and the Bangladesh National Strategy for Maternal Health (2019–2030). We have also added a brief policy-oriented statement in the conclusion section highlighting implications for national antenatal nutrition programming.
